# Aminolysis as a surface functionalization method of aliphatic polyester nonwovens: impact on material properties and biological response†

**DOI:** 10.1039/d2ra00542e

**Published:** 2022-04-11

**Authors:** Oliwia Jeznach, Dorota Kołbuk, Mateusz Marzec, Andrzej Bernasik, Paweł Sajkiewicz

**Affiliations:** Institute of Fundamental Technological Research, Polish Academy of Sciences Pawinskiego 5B 02-106 Warsaw Poland psajk@ippt.pan.pl; AGH University of Science and Technology al. Adama Mickiewicza 30 30-059 Cracow Poland

## Abstract

It is reported in the literature that introducing amino groups on the surface improves cellular behaviour due to enhanced wettability and the presence of the positive charge. In this work, electrospun fibers were subjected to aminolysis under various conditions to investigate the impact of reaction parameters on the concentration of free NH_2_ groups, change of fiber properties, and the response of L929 cells. Three types of electrospun nonwovens obtained from poly(caprolactone) (PCL), poly(l-lactide-*co*-caprolactone) (PLCL) 70 : 30 and poly(l-lactide) (PLLA) were investigated. For all polymers, the concentration of NH_2_ groups increased with the diamine concentration and time of reaction. However, it was observed that PCL fibers require much stronger conditions than PLCL and PLLA fibers to reach the same level of introduced amine groups. X-ray photoelectron spectroscopy results clearly demonstrate that an aminolysis reaction is not limited to the surface of the material. Gel permeation chromatography results support this conclusion indicating global molecular weight reduction. However, it is possible to reach a compromise between the concentration of introduced amine groups and the change of mechanical properties. For most of the investigated conditions, aminolysis did not significantly change the water contact angle. Despite this, the change of L929 and MG63 cell shape to being more spread confirmed the positive effect of the presence of the amine groups.

## Abstract 2

Aminolysis treatment improves L929 cell–scaffold interaction. It is possible to reach compromise between the concentration of NH_2_ groups and mechanical properties change.

## Graphical abstract

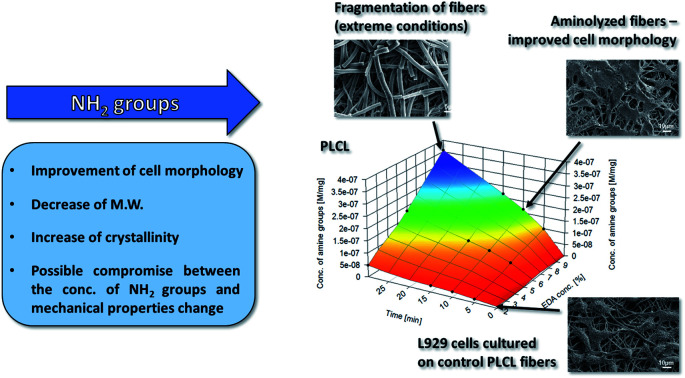

## 1. Introduction

In recent years there has been a growing interest in the modification of material surface properties in biomaterials science. There is a big advantage in modulating material surface without altering bulk properties since cell–material interaction mainly refers to the interface.^1^ Scaffolds, drug delivery systems, and implants are modified in different ways depending on the form and chemistry of the material and application site. In the case of polymer scaffolds commonly used methods are plasma treatment,^2,3^ wet chemistry (hydrolysis, aminolysis),^4^ layer by layer technique,^5^ surface graft polymerization,^6^ coating with inorganic particles^7,8^ and biomolecule immobilization.^9,10^ Most frequently, these modifications concern changing wettability, surface energy, topography, or surface elasticity or introducing charged groups or bioactive motives. These features can influence the body's response to biomaterials *via* determining protein adsorption (specificity, adsorption rate, protein conformation, exposure or not of the cell-binding region)^11–14^ and then modulating cell adhesion and their functioning, *e.g.* secretion and deposition of extracellular matrix. Several recent reviews discuss in details findings in impact of material surface properties on cells behaviour and present updated conclusion and future perspectives.^1,15,16^

In this study, we investigated aminolysis as a surface modification method of electrospun fibers made of three types of aliphatic polyesters used commonly in tissue engineering: poly(caprolactone), poly(l-lactide-*co*-caprolactone) and poly(l-lactide). Polymers differ from each other in the ratio of the ester to alkyl groups, crystallinity, and glass transition temperature, which could be essential from the reaction perspective.^17^ All obtained nonwovens have a hydrophobic nature.

The reaction between polyesters and diamines is frequently used as an effective method for introducing NH_2_ groups on the scaffold surface.^18,19^ Its application is not limited to tissue engineering. Aminolysis is used in the textile industry to enhance fibers dyeing ability.^20^ Improved adsorption of dyes by amine-functionalized fibers is also investigated for the removal of pollutants present in water.^21^ Another studied application is improving the interfacial adhesion between aminolyzed fibers and polymer matrixes.^22^ It is worth mention that aminolysis can be used for the degradation of PET fibers and bottles waste.^23,24^

**Scheme 1:**
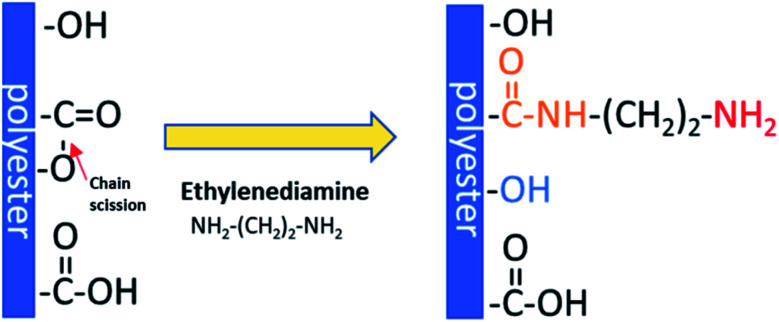
Mechanism of aminolysis reaction.

Aminolysis proceeds *via* a nucleophilic attack on the carbonyl carbon of polyester.^25^ One amino group reacts with the ester group forming an amide bond and leading to chain scission, while another one remains free. Moreover, hydroxyl groups are also created as a result of chain-breaking (Scheme 1). Aminolysis can be exploited as a single-step approach to enhance cell response as a result of improved wettability and/or presence of positive charge.^4,6,26^ On the other hand, the aminolysis technique is frequently used as an intermediate step before further physical or chemical immobilization of biomacromolecules.^27,28^ There are also other approaches to introduce free amine groups on the material surface. One of them is plasma treatment using ammonia (NH_3_) or nitrogen (N_2_) gases, or amine monomers with most common exploited allylamine.^29^ Plasma treatment is a cost-effective and relatively simple method. However, generation of active species inside of the scaffold and alteration of modifying depth have to be taken into consideration.^30^ Another method is incorporation of amine-functionalized PEG-dendrimers,^31^ poly(ethyleneimine),^32^ which is rich in primary amine groups, using *e.g.* EDC/NHS crosslinking or poly(dopamine), which is easily deposited by direct immersion of scaffold in dopamine solution.^33^ However, it is rather a way to further conjugation of biomolecules with incorporated free –NH_2_ groups as these substances are known for suppression of protein adsorption and some cell adhesion, mainly due to its high hydrophilicity. Hence, these coatings are exploited in manufacturing of hemocompatible scaffolds.^34–36^ Aminolysis, which is kind of wet chemical method, is a simple way to homogenously introduce primary amine groups on the scaffold surface without complete change of surface chemistry.

It is reported that protein adsorption on hydrophobic surfaces can lead to their denaturation, which can make them unable to bind cells.^37^ It was experimentally found that too high hydrophilicity could also be unfavourable for cell attachment.^38^ Hence, it appears that moderate wettability provides the proper amount of adsorbed proteins as well as unchanged conformation, which results in good cell response.^39^ From this perspective, it is important that aminolysis can cause an increase of wettability.^40^ Considering charge of surface, it was experimentally proved that a positively charged surface has a beneficial impact on cell behaviour, most probably due to electrostatic interactions allowing effective adhesion of negatively charged proteins present in serum, as well as direct interaction with the charged surface of the cell membrane.^41,42^ Liamas E. *et al.* used amine surface (–NH_3_^+^) in computational modelling of fibronectin adsorption and showed that in contrast to uncharged surface, protein adsorption on positively charged surface is relatively rapid and site-specific.^13^

Although diamine-based surface modification of polyesters was fundamentally studied by others, the vast majority of experimental results referred to solvent-casted membranes.^22,43–47^ Currently, aminolysis is frequently used as direct or indirect functionalization of ECM-mimicking electrospun fibers^22,48–53^ and there is no comprehensive study in the literature that concerns the optimization of this reaction for various polyester fibers.

In the previous paper, we discussed reasons for different susceptibility of electrospun fibers and solvent-casted films to aminolysis.^15^ In this work, the aminolysis of three types of aliphatic polyester fibers is investigated more detailed in a wide range of conditions. We focus on fundamental characterization and comparison of materials in terms of surface characterization including XPS and amino groups concentration, impact of the modification on fibers morphology, molecular weight, wettability, mechanical properties and crystallinity, as well as interaction of materials with L929 and MG63 lines cells.

## 2. Experimental

### 2.1. Materials

Three types of polyesters were used: PCL (Sigma-Aldrich, USA, 
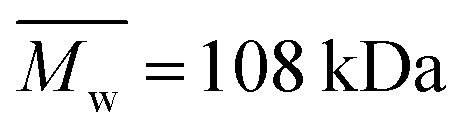
, 
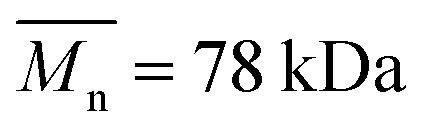
, *T*_g_ = −60 °C), PLCL 70 : 30 (Resomer® LC703S, Evonik, Germany, 
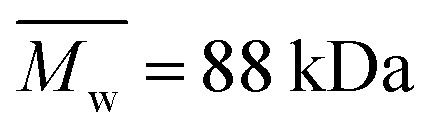
, 
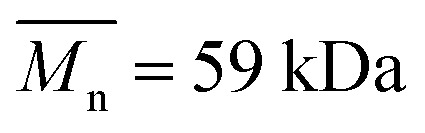
, inherent viscosity = 1.3–1.8 dl g^−1^, *T*_g_ = 32–42 °C) and PLLA (PL49, Corbion, Netherlands, 
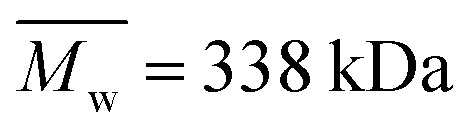
, 
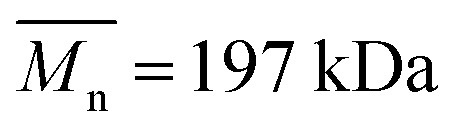
, inherent viscosity = 4.9 dl g^−1^, *T*_g_ = 58 °C). Solvents used in electrospinning process—acetic acid (AA) (purity degree 99.5%), formic acid (FA) (purity degree ≥ 98%), and hexafluoroisopropanol (HFIP) (purity degree 98.5%) were purchased from Poch (Poland), Sigma-Aldrich (USA), and Iris Biotech GmbH (Germany), respectively. Ethylenediamine (EDA) (purity degree 99.5%), isopropanol (purity degree 99.8%), ninhydrin (purity degree 99%), ethanol (purity degree 99.8%), glutaraldehyde (conc. 25%) and tetrahydrofuran (THF) (for HPLC) were purchased from Chempur (Poland). Chloroform (LiChrosolv®, for liquid chromatography) was procured from Sigma-Aldrich (USA). For all polymers, 
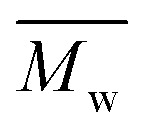
 and 
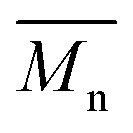
 were determined experimentally.

### 2.2. Preparation of electrospun fibers

Three types of nonwovens were fabricated using electrospinning equipment (Bioinicia, Spain) in horizontal mode. Solutions (15% w/w solution of PCL in a mixed solution of acetic acid and formic acid at 9 : 1 ratio, 7% w/w PLCL solution in HFIP, and 3.5% w/w solution of PLLA in HFIP) were pumped through two stainless steel needles with a speed of 6 ml h^−1^ (3 ml h^−1^ per needle). Other process parameters were: distance needle-collector: 15 cm, collector rotating speed: 300 rpm, voltage applied to collector: −2 kV, voltage applied to needles: PCL: 13–15 kV, PLCL: 12–14 kV, PLLA: 12–14 kV, temperature and humidity conditions: PCL: 24 °C, 40%, PLCL: 38 °C, 40%, PLLA: 24 °C, 40%, volume of solution used to obtain 12 cm × 31 cm nonwoven – PCL: 13 ml, PLCL: 18 ml, PLLA: 39 ml.

### 2.3. Aminolysis treatment

Samples of 5.5 × 5.5 cm size were immersed in 80 ml of ethylenediamine in isopropanol solution. Solutions concentrations were: 10, 20, 30% w/v for PCL and 2, 6, 10% w/v for PLCL and PLLA. Time points were: 30 min, 6 h, 24 h, 72 h for PCL and 5 min, 10 min, 15 min, 30 min for PLCL and PLLA. The temperature of the reaction was set at 30 °C. The process was conducted in an orbital shaker-incubator. After that, aminolysis samples were washed three times in deionized water and dried under vacuum overnight. Sample designations were set as the name of polymer_EDA conc._reaction time, *e.g.* PCL_20%_72 h or as the name of polymer_Control for control, unmodified samples.

### 2.4. Surface characterization

#### 2.4.1. Detection and quantification of amino groups

Ninhydrin colorimetric test was used to quantify the concentration of amine groups grafted during aminolysis. Three samples of weight in the range of 10–30 mg, depending on the type of polymer and reaction conditions, were used. Each sample was put into a glass vial, and 0.5 ml of 2% w/v ninhydrin in ethanol/acetic acid (49 : 1 v/v) solution was added. Vials were heated for 10 min using a hot plate set at 100 °C. After that, 1 ml of isopropanol (in the case of PLCL and PLLA) or 0.5 ml of isopropanol and 0.5 ml of dioxane (in the case of PCL) were added. The absorbance of tested solutions was measured at 570 nm. The concentration of amine groups was calculated on the basis of the calibration curve obtained for a known concentration of ethylenediamine in isopropanol solution. In the case of PCL, the volume of dissolved material was included in the calculation. Results were presented as a concentration of amino groups in moles per mg of fibrous sample.

#### 2.4.2. XPS analysis

Five chosen samples were analyzed using XPS: PLCL_Control, PLCL_2%_15 min, PLCL_6%_15 min, PLCL_6%_30 min and PLCL_10%_15 min. XPS analyses were performed using a scanning XPS system (PHI VersaProbe II, USA) equipped with monochromatic Al Kα (1486.6 eV) radiation. The power X-ray source was about 25 W. X-rays beam were focused to a 100 μm spot which determined the area of analysis. The measurement time of the spectrum of one element was about 7 minutes. A dual beam charge compensation with 7 eV Ar^+^ ions and 1 eV electrons were used to maintain a constant sample surface potential. All XPS spectra were referenced to the unfunctionalized, saturated carbon (C–C) C1s peak at binding energy (BE) equal to 284.8 eV. Concentration of elements and their chemical sates on various depth of PLCL fiber were measure by sputtering with Argon Gas Cluster Ion Beam (Ar-GCIB). Sputtering parameters were set to 10 kV beam voltage and 30 nA current with approx. 4000 argon atoms per cluster. The size of the sputtered area was set to 3 × 3 mm. During the sputtering Zalar rotation was applied. The sputtering rate was determined in a separate experiment using a 450 nm thick PLCL layer and was set at 4.1 nm min^−1^. The analysis of the fiber composition was performed on the surface and after consecutive 15 minute sputtering cycles.

### 2.5. SEM imaging

Scanning electron microscopy (SEM, Jeol JSM-6010PLUS/LV InTouchScope™) was used for sample imaging. Before imaging, samples were coated with gold. The acceleration voltage was in the range of 7–10 kV.

### 2.6. Wettability

The water contact angle (WCA) was determined for aminolyzed samples using goniometer Data Physics OCA 15EC (Germany). The dosing volume and dosing rates were 1 μl and 2 μl s^−1^, respectively. Measurements were repeated 5 times for each sample.

### 2.7. Change of average molecular weight

Change of weight average molecular weight 
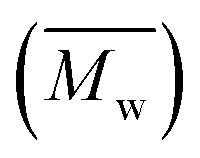
, number average molecular weight 
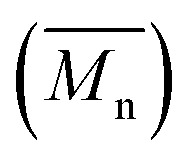
, and polydispersity index (PI) were measured using gel permeation chromatography (GPC, Shimadzu). PCL and PLCL samples were solubilized in THF at a concentration of 3 mg ml^−1^. PLCL granules and all PLLA samples were solubilized in chloroform due to poor solubility in THF at a concentration of 1 mg ml^−1^, respectively. THF was used as a mobile phase. The temperature was set at 40 °C. All solutions were filtered by 0.22 μm syringe filters (Biosens). THF flow rate was equal to 1 ml min^−1^. Polystyrene standards with 
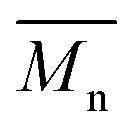
 from 3470 Da to 2 520 000 Da (PSS Polymer Standards Service GmbH) were used to create a calibration curve. GPC equipment consisted of a pump, column (Phenogel™ 5 μm 10e5Å, Phenomenex), refractive index detector (RID-20A, Shimadzu) and LabSolutions GPC software. In the case of PLLA samples, the calculation of 
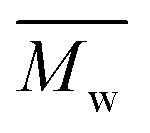
 and 
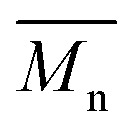
 were conducted using OriginPro 8.0 and PeakFit 4.12 software due to disturbance resulting from incomplete solubility of PLLA in chloroform. The Mark–Houwink equation parameters were as follow *α* = 0.7, *K* = 14.1 × 10^−5^ dl g^−1^ for polystyrene standard polymer, *α* = 0.786, *K* = 13.95 × 10^−5^ dl g^−1^ for PCL according to Sinnwell S. *et al.*,^54^*α* = 0.68, *K* = 4.84 × 10^−4^ dl g^−1^ for PLCL and *α* = 0.65, *K* = 0.001 for PLLA according to Nuuttila M. *et al.*^55^

Sets of the two aminolyzed samples for each type of polymer were chosen for further analysis including mechanical testing, WAXS analysis and cell response. The first sample was selected as having a lower concentration of amine groups ((1.42–3.18) × 10^−8^ mol mg^−1^) and the second as having a higher concentration of amine groups ((1.65–1.75) × 10^−7^ mol mg^−1^).

### 2.8. Mechanical testing

Uniaxial testing machine Lloyd EZ-50 (USA) was used to determine Young's modulus, stress at break and strain at break. Three samples (5 × 40 mm, testing area: 5 × 20 mm) for each type of material were tested. Thickness of each sample was measured with a thickness gauge. Machine was equipped with handles for thin and delicate samples with a 50 N load cell. Cross-head speed was set at 5 mm min^−1^.

### 2.9. WAXS analysis

Wide angle X-ray scattering (WAXS) was applied for analysis of the supermolecular structure. WAXS measurements were performed using Bruker D8 Discover diffractometer (Mannheim, Germany) with CuKα radiation operated at a voltage of 40 kV, and a current of 20 mA. All measurements were performed in reflection mode, using Goebel optics for beam formation: a 0.6 mm slit and Soller collimator. Highly sensitive Lynx Eye 1-D silicon strip detector was used. The range of diffraction angle, 2*θ*, was between 5° and 35°, with a step of 0.01° and a time of data accumulation at angular point of 0.2 s. The “empty” scan without a sample was subtracted and the default function of subtracting background was applied. Then, the WAXS profiles were deconvoluted numerically using PeakFit software assuming Pearson VII and Gauss functions for the crystal diffraction peaks from the amorphous halo, respectively. The degree of crystallinity was determined as the ratio of the area of all the crystalline diffraction peaks to the overall area of the profile.

### 2.10. ATR-FTIR analysis

Surface molecular structure of samples was analyzed using attenuated total reflectance Fourier transform infrared (ATR-FTIR) spectrometer Bruker Vertex 70 (Mannheim, Germany). Absorbance mode in 4000–400 cm^−1^ range was applied. The resolution of measurement was equal to 2 cm^−1^.

### 2.11. Cell response

Mouse fibroblast cells (L929, Sigma Aldrich) and osteoblast-like cells (MG63, Sigma Aldrich) were chosen to investigate cell response. For L929 cells culture, Dulbecco's Modified Eagle Medium (DMEM, Thermo Fisher Scientific) was supplemented with fetal bovine serum (FBS, Thermo Fisher Scientific) to the concentration of 10% v/v and penicillin/streptomycin (Thermo Fisher Scientific) to the concentration of 1% v/v. For MG63 cells culture, 1% v/v of gentamicin (Thermo Fisher Scientific) was added to the media.

Cells were cultured in an incubator at 40 °C in a 5% CO_2_. Samples of nonwovens were sterilized using 70% – solution of ethanol in water and 30 min-exposure to UV light on each side. Samples were placed in a 48-well culture plate – three samples for cell metabolic activity test, and two samples for SEM observation for each type of material. 2 × 10^3^ cells were seeded per well. Cells were cultured in an incubator for 5 days.

PrestoBlue™ (Thermo Fisher Scientific) test was chosen to evaluate cell metabolic activity. After removing the culture medium, each sample was rinsed with phosphate-buffered saline (PBS, Thermo Fisher Scientific). Then 180 μl of PBS and 20 μl of PrestoBlue™ reagent in the PBS solution was added to each well. PrestoBlue reagent was also added to pure PBS as a control. The plate with samples was further put into an incubator for 40 min to enable reaction. After that, 100 μl of each solution was placed in a 96-well plate. Emission of light was measured using Fluoroskan Ascent (Thermo Scientific) at an excitation light wavelength of 530 nm and emission light wavelength of 620 nm. Results were then calculated to percentage metabolic activity using tissue culture polystyrene as a 100% control.

Samples used for SEM observation were gently rinsed with PBS twice and then fixed with 2.5% glutaraldehyde/PBS overnight. Samples were further dehydrated using ethanol in water solution series (30, 40, 50, 60, 70, 80, 90, 100 (×2)% v/v), hexamethyldisilazane (HDMS, Sigma Aldrich)/ethanol solutions (1 : 2 and 2 : 1 v/v), and pure HDMS. Each ethanol solution was removed after 20 minutes, and HDMS was left overnight.

Fluorescence microscopy imaging was performed after immunohistochemical staining. Cells were washed once with phosphate buffered saline (PBS), fixed in 4% paraformaldehyde, kept in 0.1% Triton-X for 5 min and washed again with PBS. Then the actin skeleton and nucleus were stained with ActinGreen™ and NucBlue™ reagent (Thermo Scientific), respectively. All samples in comparison to TCP (Tissue Culture Plastic) were illustrated using a fluorescent microscope (Leica DMI3000B, Leica Microsystems).

### 2.12. Statistical analysis

One-way analysis of variance (ANOVA) followed by NIR *post hoc* test, or Kruskal–Wallis one-way analysis of variance was performed on WCA, stress and strain at break, and cell metabolic activity data test using Statistica 13 software. Significance was set at *p* < 0.05.

Results are presented as the mean value ± SD.

## 3. Results

**Fig. 1:**
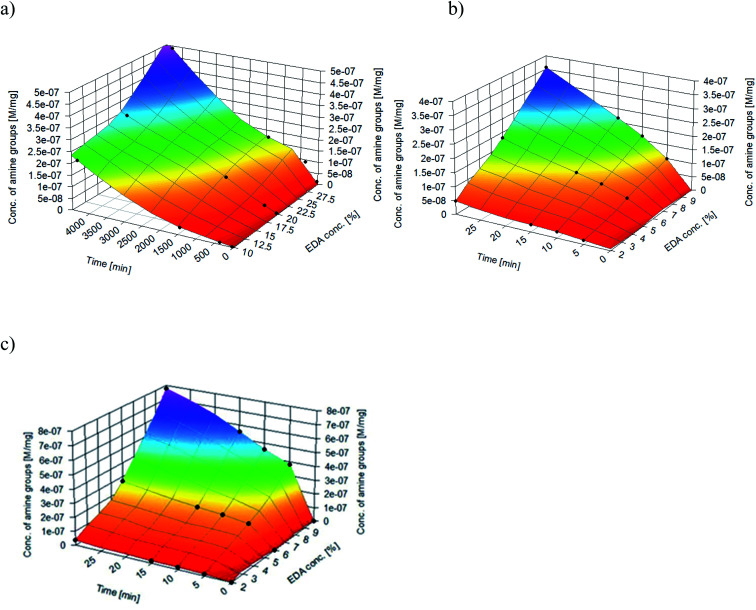
Concentrations of NH_2_ groups on the surface of (a) PCL, (b) PLCL, (c) PLLA fibers subjected to aminolysis at 30 °C.

Fig. 1 shows the results of the ninhydrin test, which allowed to measure NH_2_ groups introduced to samples subjected to aminolysis. The first observation is that the concentration of NH_2_ groups on the surface increases for all polyesters with ethylenediamine concentration and reaction time. For PLCL and PLLA the same conditions were applied, and it was observed that PLLA fibers were more susceptible to aminolysis. For instance, in the conditions of 10% EDA concentration and 30 min time of reaction, the concentration of NH_2_ groups for PLCL was (3.57 ± 0.01) × 10^−7^ mol mg^−1^ and for PLLA (7.75 ± 0.26) × 10^−7^ mol mg^−1^. The results obtained for PCL fibers are even more contrasting. In the case of this polyester, there was a need to use much higher concentrations of EDA and reaction times, which was concluded on the basis of preliminary studies.^17^ However, even for extreme conditions (30%, 72 h) of PCL aminolysis, the concentration of NH_2_ groups is still lower than in the case of the most reacted PLLA.

**Fig. 2:**
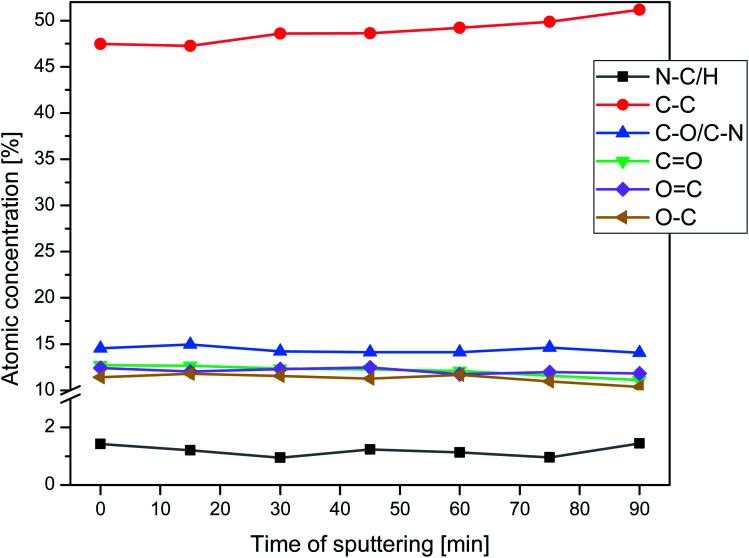
Atomic concentration of the detected chemical bonds for PLCL_10%_15 min sample *vs.* sputtering time.

**Fig. 3:**
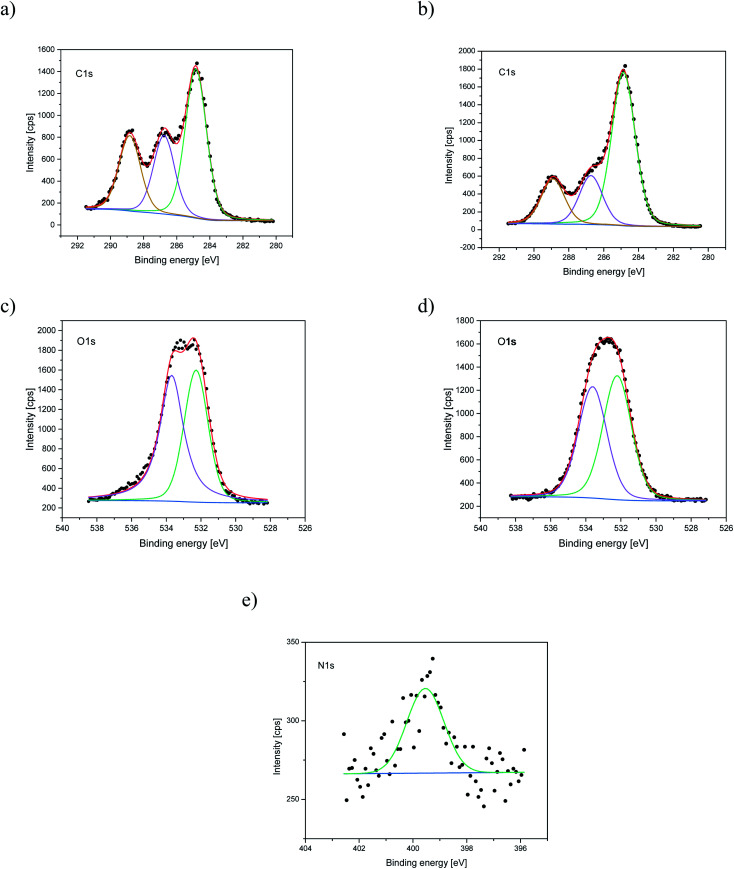
XPS spectra of the C1s, O1s, N1s for PLCL_Control (a, c) and PLCL_10%_15 min (b, d, e) samples.

Nitrogen detection using the XPS method was possible for the PLCL_10%_15 min sample. The concentration was measured to be equal to 1.1 at%. In the case of other aminolyzed samples, nitrogen atomic concentration was too low to be detected by XPS. Fig. 2 presents a depth profile of the atomic concentration of the detected chemical bonds for the PLCL_10%_15 min. Taking into account sputtering rate, time of 90 minutes corresponds to the depth of about 370 nm. There were no significant changes in atomic concentration of all chemical bonds on the studied depth. Fig. 3 shows C1s, O1s, and N1s spectra for PLCL_Control and PLCL_10%_15 min. Table 1 presents atomic concentration and the relative intensity of the C1s peaks measured by XPS on the surface of PLCL_Control and PLCL_10%_15 min. A detailed analysis of the C1s spectra allowed to determine the concentration of the C–C (BE: 284.8 eV), C–O/C–N (BE: 286.6 eV), and C=O (BE: 288.8 eV) bonds. Whereas the C–O and C–N bond energies differ slightly and also that the nitrogen concentration is low, both chemical states have been fitted with one peak.

**Table 1:** The atomic concentration and the relative intensity of the C1s peaks measured by XPS on the surface of PLCL_Control and PLCL_10%_15 min (before sputtering)

Sample	Concentration of amine groups [mol mg^−1^]	Atomic concentration [%]			Relative intensity [%]		
		C	O	N	C–C	C–O	C=O
						C–N	
PLCL_Control	0	69.6	30.4	—	48.1	25.6	26.3
PLCL_10%_15 min	(2.16 ± 0.15) × 10^−7^	73.1	25.8	1.1	61.1	19.6	19.3

Additionally, we performed ATR-FTIR analysis. We observed a small signal around 1530 cm^−1^ for aminolyzed samples, the intensity of which increases with the time of aminolysis. According to the literature, this signal can be attributed to the amide II band originating from N–H stretch vibrations in primary amines and from a mixed vibration of N–H bending and C–N stretching in secondary amides.^56^ It corresponds well with the situation where one of the diamine ends is anchored to the polymer substrate, mimicking thus the existence of primary amine with only one free end. The FTIR data were attached as ESI (Fig. S1†).

**Fig. 4:**
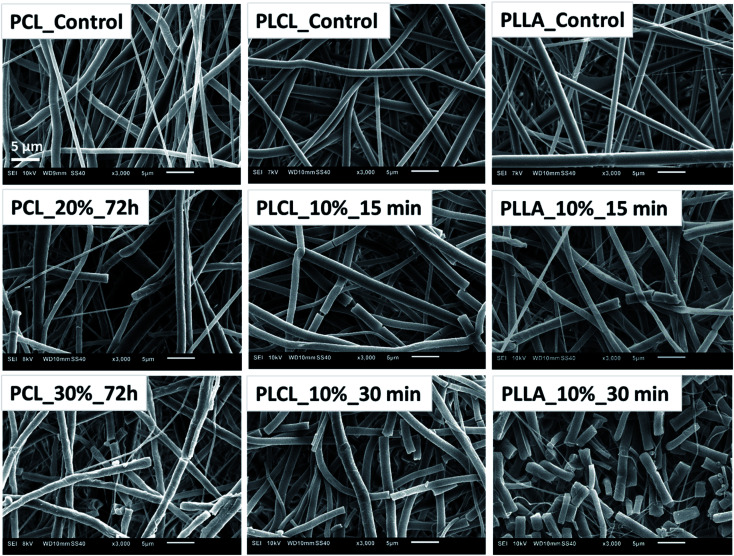
SEM images of fibers breaking as a result of aminolysis under most aggressive conditions.

The aminolysis process did not cause the visible change of fibers morphology in most of the samples. However, for each type of polyester, fractured fibers were observed for the most aggressive aminolysis conditions corresponding to the highest concentration of amine groups (Fig. 4). Fragmented fibers were observed for PCL_20%_72 h, PCL_30%_72 h, PLCL_10%_15 min, PLCL_10%_30 min, PLLA_10%_15 min and PLLA_10%_30 min. The intensity of fragmentation was different depending on the type of polymer.

**Fig. 5:**
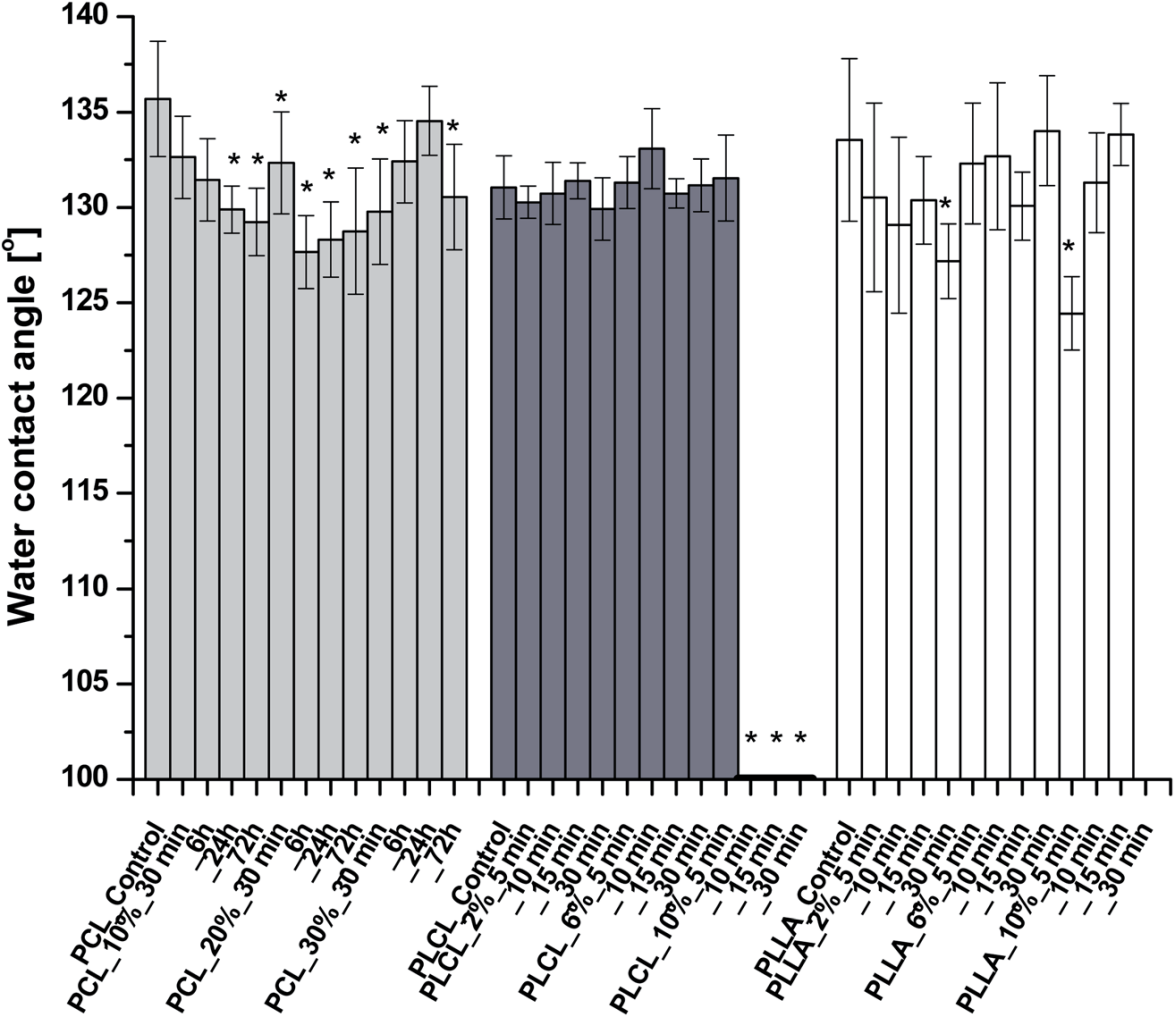
Water contact angles of aminolyzed samples. WCAs of three last PLCL samples were equal to 0°. No data for PLLA_10%_30 min due to sample degradation. Statistically significant difference between control and modified sample was marked as * (*p* < 0.05).

The results of WCA measurements are shown in Fig. 5. Despite the hydrophilic nature of amine groups, there is no significant change of WCA for most of the functionalized samples. Slight differences in water contact angle, which were observed, can originate from the nature of fibrous samples like variability in density and coverage of polymer fibers on the substrate, leading to masking of the wettability itself. In the case of PCL, the largest WCA decrease was only 8 degrees, for PLLA – 9 degrees. PLCL samples also remained hydrophobic with almost the same WCA as a native sample, except the three samples treated most aggressively, which manifest complete hydrophilicity in time of 293.10 ± 78.71 s, 25.08 ± 8.34 s, and 7.17 ± 3.08 s, respectively. It is worth mentioning that the fibers in two of these samples were strongly fragmented.

**Fig. 6:**
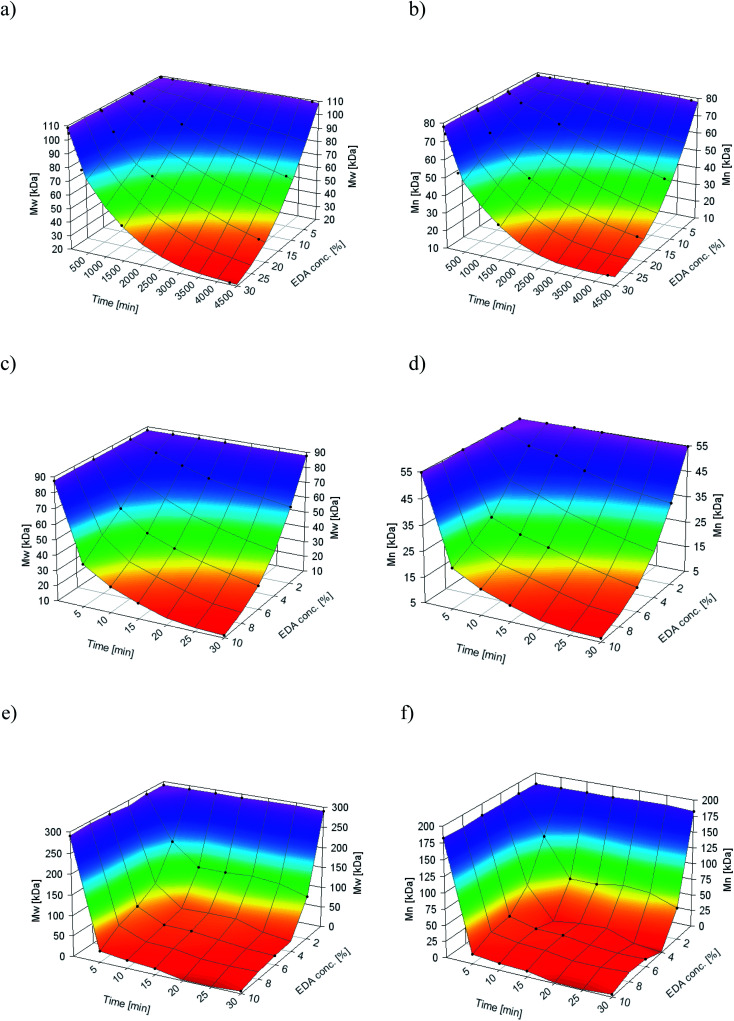
Change of 
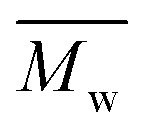
 of (a) PCL, (c) PLCL, and (e) PLLA, and 
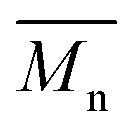
 of (b) PCL, (d) PLCL, and (f) PLLA *vs.* EDA concentration and reaction time.

As aminolysis is a kind of degradation due to the cleavage of ester bonds, the study of molecular weight change is an important input into its investigation. Fig. 6 shows 3D plots of weight and number average molecular weights, as a function of time and diamine concentration for all investigated polymers. Additionally, Table 2 contains source molecular weight data together with polydispersity index and corresponding amine concentration. Comparing three studied nonwovens, it is evident that the rate of drop in molecular weight is the slowest in the case of PCL and the fastest for PLLA.

**Table 2:** Conc. of amine groups, molecular weights, and polydispersity of electrospun fibers

Sample	Conc. of NH_2_ groups [mol mg^−1^]	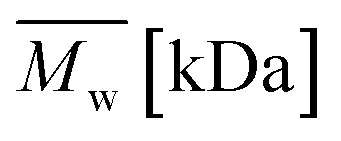	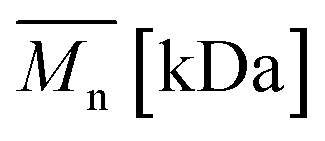	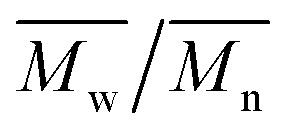	Sample	Conc. of NH_2_ groups [mol mg^−1^]	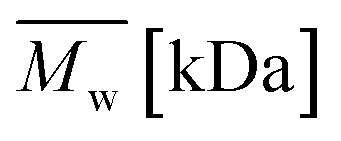	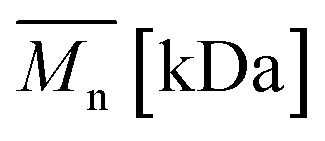	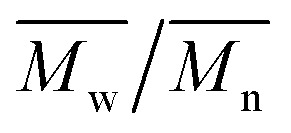
PCL_Control	0	108.8	78.1	1.39	PLCL_6%_15 min	(1.09 ± 0.08) × 10^−7^	38.8	23.0	1.69
PCL_10%_30 min	(1.81 ± 0.62) × 10^−9^	107.9	77.0	1.40	PLCL_6%_30 min	(1.79 ± 0.13) × 10^−7^	25.5	14.4	1.77
PCL_10%_6 h	(8.76 ± 0.38) × 10^−9^	104.0	72.7	1.43	PLCL_10%_5 min	(9.86 ± 0.44) × 10^−8^	37.8	20.7	1.82
PCL_10%_24 h	(3.18 ± 0.31) × 10^−8^	91.8	64.3	1.43	PLCL_10%_10 min	(1.65 ± 0.15) × 10^−7^	26.7	15.2	1.76
PCL_10%_72 h	(2.18 ± 0.43) × 10^−7^	66.9	44.1	1.52	PLCL_10%_15 min	(2.16 ± 0.15) × 10^−7^	20.0	11.4	1.75
PCL_20%_30 min	(6.28 ± 1.00) × 10^−9^	108.2	77.1	1.40	PLCL_10%_30 min	3.57 × 10^−7^	11.6	6.5	1.80
PCL_20%_6 h	(3.01 ± 0.66) × 10^−8^	94.3	65.6	1.44	PLLA_Control	0	290.4	182.4	1.59
PCL_20%_24 h	(1.18 ± 0.03) × 10^−7^	66.6	44.1	1.51	PLLA_2%_5 min	(1.36 ± 0.13) × 10^−8^	182.2	121.0	1.51
PCL_20%_72 h	(3.04 ± 0.31) × 10^−7^	35.9	23.3	1.54	PLLA_2%_10 min	(1.78 ± 0.24) × 10^−8^	129.2	60.9	2.12
PCL_30%_30 min	(1.28 ± 0.53) × 10^−8^	104.9	74.3	1.41	PLLA_2%_15 min	(1.95 ± 0.24) × 10^−8^	128.4	61.1	2.10
PCL_30%_6 h	(9.30 ± 1.32) × 10^−8^	80.1	53.9	1.49	PLLA_2%_30 min	(3.66 ± 0.49) × 10^−8^	106.5	36.7	2.17
PCL_30%_24 h	(1.75 ± 0.20) × 10^−7^	45.5	29.7	1.53	PLLA_6%_5 min	(1.49 ± 0.12) × 10^−7^	74.8	32.4	2.31
PCL_30%_72 h	(5.05 ± 0.81) × 10^−7^	21.4	14.7	1.45	PLLA_6%_10 min	(1.75 ± 0.22) × 10^−7^	42.7	21.8	1.96
PLCL_Control	0	87.5	55.0	1.59	PLLA_6%_15 min	(1.89 ± 0.09) × 10^−7^	40.6	20.6	1.97
PLCL_2%_5 min	(1.42 ± 0.08) × 10^−8^	81.3	49.8	1.48	PLLA_6%_30 min	(2.63 ± 0.18) × 10^−7^	22.6	12.4	1.82
PLCL_2%_10 min	(2.23 ± 0.21) × 10^−8^	75.9	47.8	1.59	PLLA_10%_5 min	(3.80 ± 0.37) × 10^−8^	27.5	15.0	1.83
PLCL_2%_15 min	(2.57 ± 0.32) × 10^−8^	70.5	43.7	1.61	PLLA_10%_10 min	(4.53 ± 0.25) × 10^−8^	19.0	10.8	1.76
PLCL_2%_30 min	(4.87 ± 0.30) × 10^−8^	61.1	36.9	1.65	PLLA_10%_15 min	(5.53 ± 0.39) × 10^−8^	13.8	8.4	1.65
PLCL_6%_5 min	(5.43 ± 0.90) × 10^−8^	58.4	30.6	1.9	PLLA_10%_30 min	(7.75 ± 0.26) × 10^−8^	5.9	3.8	1.56
PLCL_6%_10 min	(8.63 ± 1.16) × 10^−8^	45.5	25.9	1.76					

**Fig. 7:**
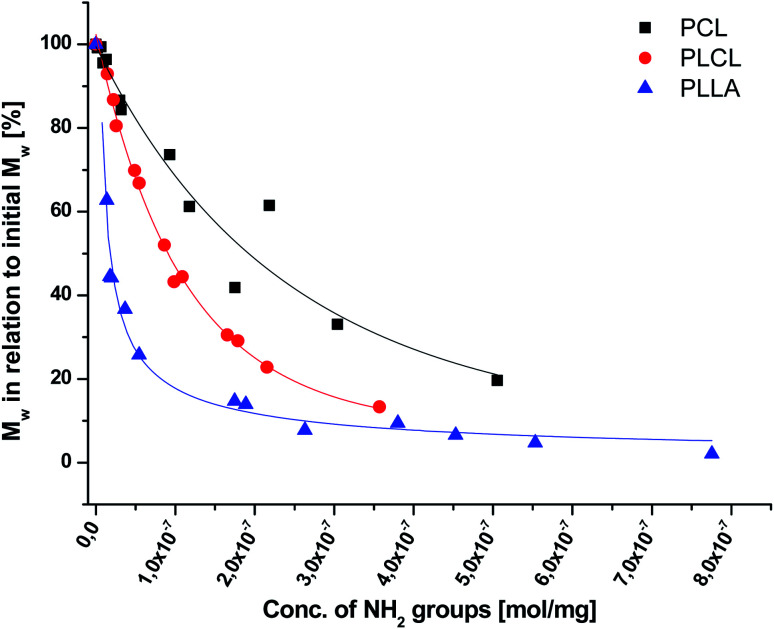
Relative change of weight average molecular weight, 
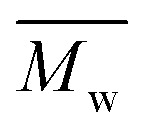
, for PCL, PLCL, and PLLA fibers *vs.* grafted NH_2_ groups concentration. For PCL and PLCL, data were fitted with exponential curves, and for PLLA with an allometric curve.

In Fig. 7, a correlation between the concentration of introduced amine groups and the molecular weight is presented. Considering the same concentration of attached amine groups, the lowest drop in 
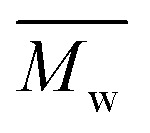
 was observed for PCL and the highest for PLLA.

**Fig. 8:**
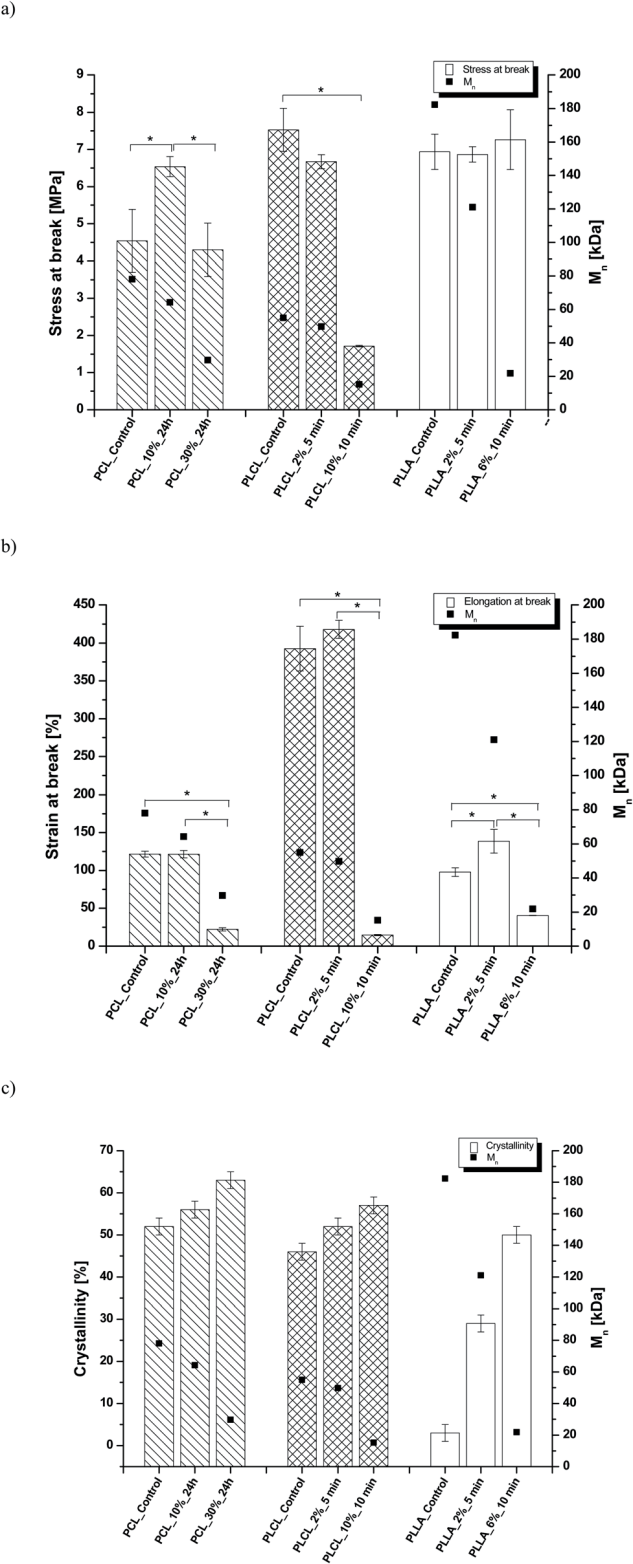
(a) Stress and (b) strain at break, and (c) crystallinity of studied fibers in the function of 
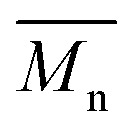
 mass. Statistically significant differences were marked as * (*p* < 0.05).

In Fig. 8 data of 
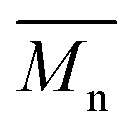
 were compiled with stress and strain at break, and crystallinity for two chosen samples of each type of fibers after mild and strong aminolysis treatment. A noticeable drop of stress at break is observed only for PLCL_10%_10 min. A significant decrease of strain at break was noticed for all samples after strong aminolysis. In general, the changes of stress at break are not correlated with 
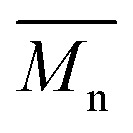
 – for instance there is a strong reduction of 
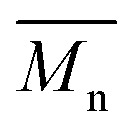
 for PLLA without any essential changes of stress at break. Regarding mechanical properties, there is a better correlation between strain at break and 
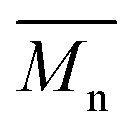
, particularly evident for the most aggressive aminolysis conditions (Fig. 8b). A decrease of number average molecular weight was clearly correlated with an increase of crystallinity. We have added exemplary WAXS profiles as ESI (Fig. S2 and S3†).

**Fig. 9:**
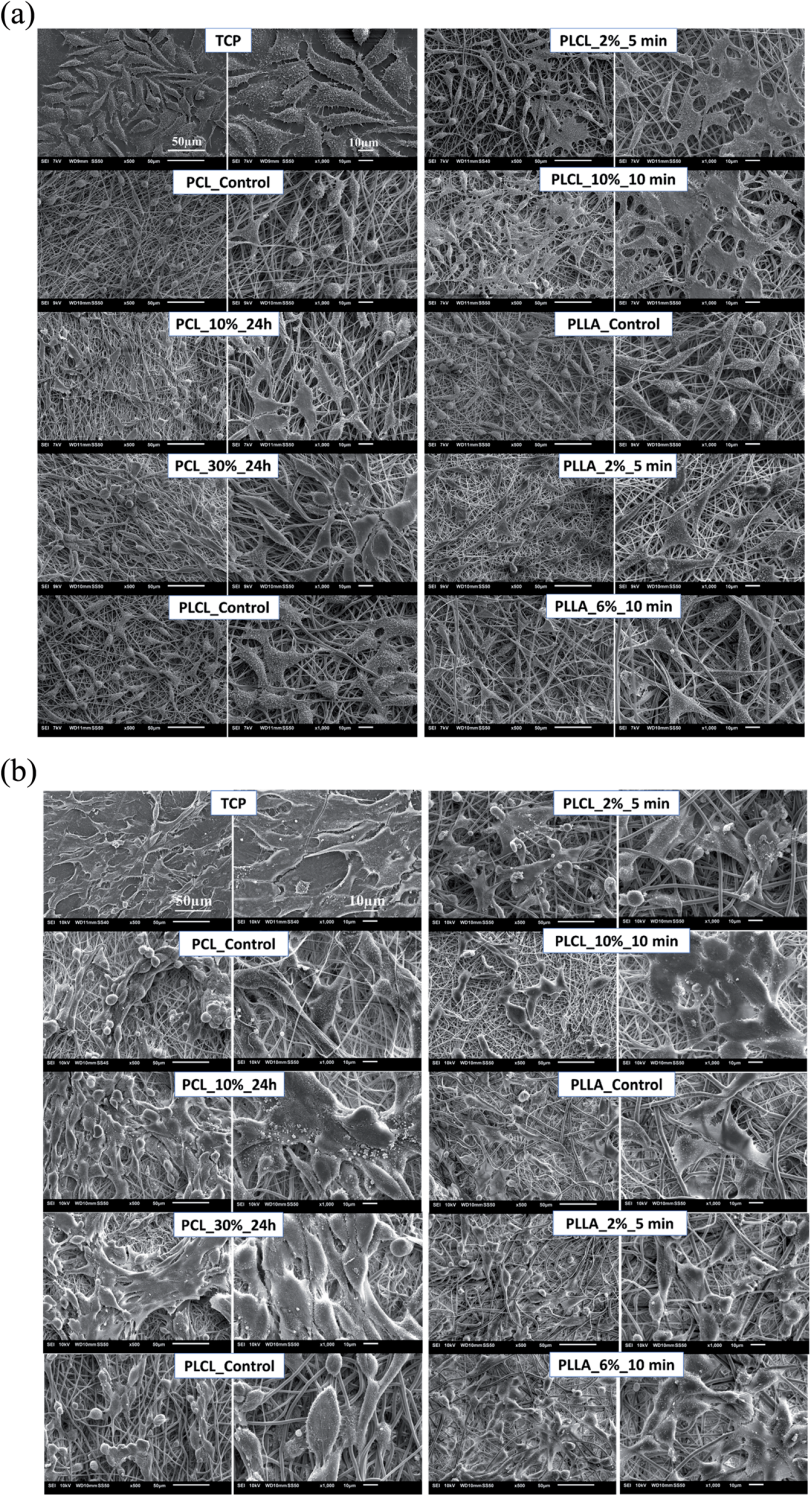
SEM images of (a) L929 cells and (b) MG63 cells cultured by 5 days on control and modified fibers and TCP.

Effect of aminolysis treatment on cell response to materials was studied on two chosen samples of each type of fibers. Fibroblast L929 cells and osteoblast-like MG63 cells were cultured on them for 5 days. In Fig. 9a, there are SEM images of L929 cells morphology. Cells seeded on unmodified (control) fibers have rather an unfavourable round shape compared to TCPS. Cells morphology has changed to be more spread in the case of all aminolyzed samples. It was especially observed in the case of PLCL_10%_10 min; however, it should be taken into account that initially, PLCL fibers promote the best cell morphology among control samples. In Fig. 9b, there are SEM images of MG63 cells morphology. For PCL and PLCL fibers after aminolysis, improvement of cell morphology is observed. In the case of PLLA fibers, control samples provide sufficient conditions for material–cells interaction. It seems that in that case aminolysis treatment causes slight deterioration of cell morphology.

**Fig. 10:**
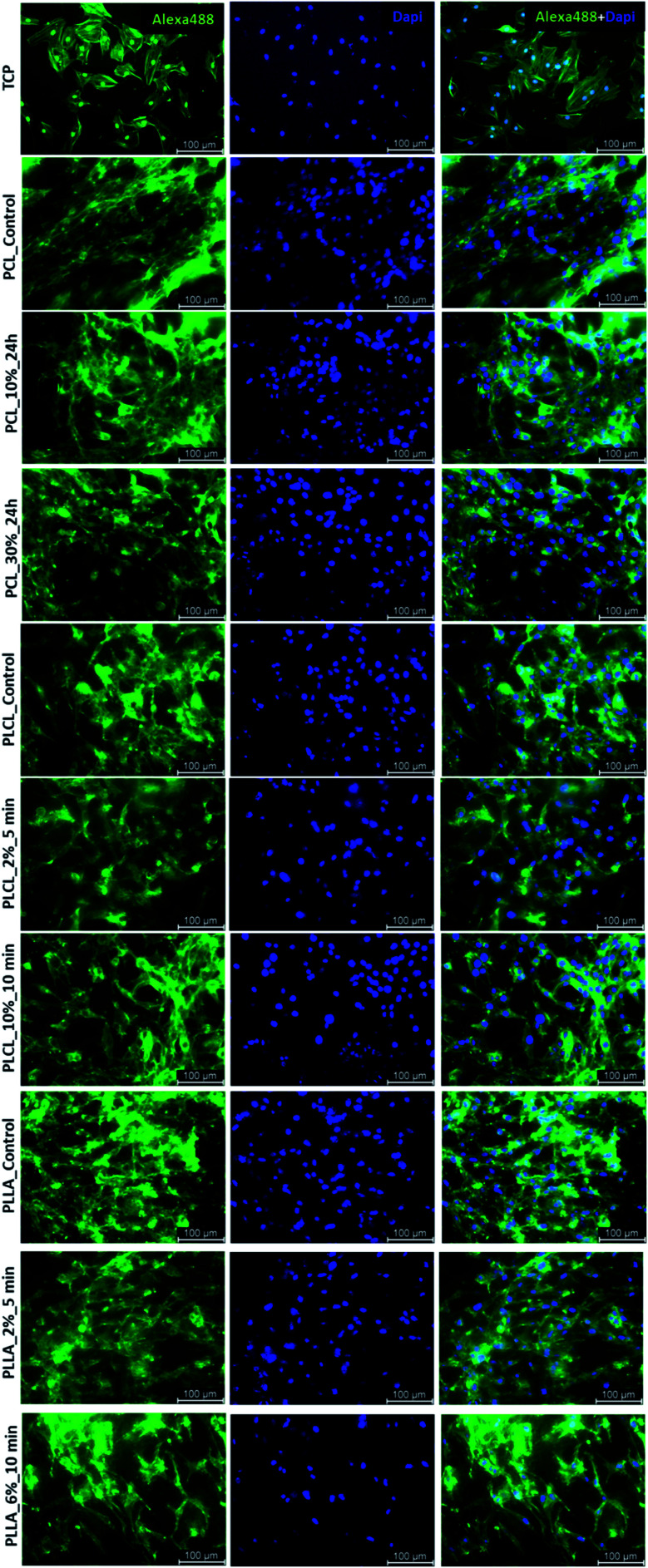
FM images MG63 cells cultured by 5 days on control and modified fibers and TCP.

In Fig. 10 there are FM images of MG63 cells cultured on studied materials. Greater spreading of actin skeleton is particularly noticeable for PCL samples after aminolysis in comparison to PCL_Control. In the case of PLCL and PLLA fibers, proper cells morphology without significant differences between samples was observed.

**Fig. 11:**
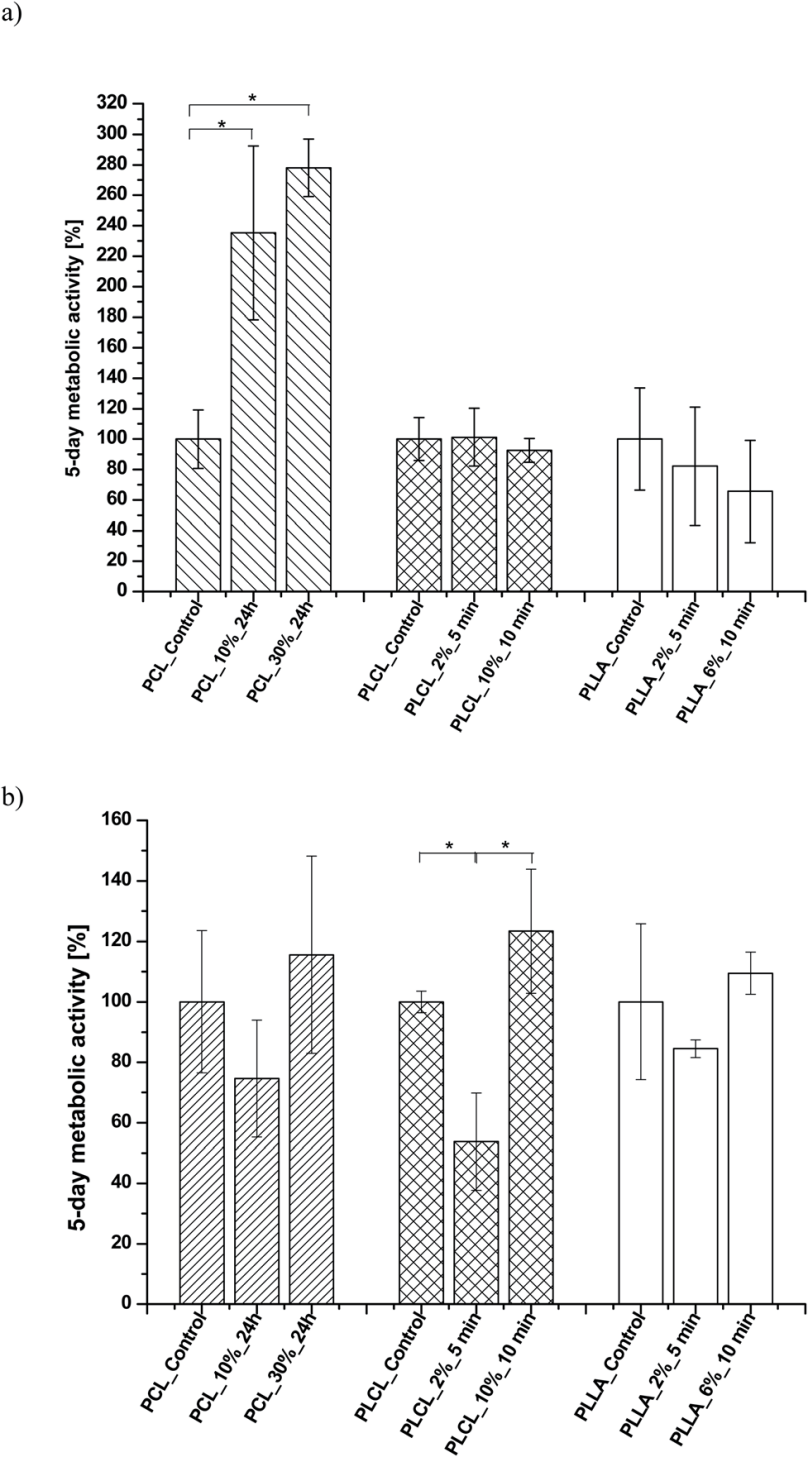
Metabolic activity of (a) L929 cells and (b) MG-63 cells after 5 days of culture on control and modified fibers. Statistically significant differences were marked as * (*p* < 0.05).

In Fig. 11a, the change of metabolic activity of L929 cells was shown. We observed gradual improvement of metabolic activity in the case of PCL samples after aminolysis. Metabolic activity increases up to 235.36 ± 56.94% and 278.08 ± 18.90% of PCL_Control value for a lower and higher concentration of amine groups, respectively. In the case of PLCL samples, both unmodified and modified, metabolic activity was on the same level, indicating that aminolysis does not change the metabolic activity of cells seeded on PLCL fibers. In the case of PLLA samples, we observed a decrease in metabolic activity for modified samples. In Fig. 11b, the change of metabolic activity of MG63 cells was shown. There is only a slight increase for samples of each polymer with higher concentration of amine groups. For samples with lower amine groups concentration, a decrease of metabolic activity was observed.

## 4. Discussion

Based on the ninhydrin test results, the susceptibility of the investigated fibers to aminolysis can be presented as PLLA > PLCL ≫ PCL. PCL fibers require a much higher concentration of EDA and time of reaction to reach the same level of attached amine groups as PLCL and PLLA fibers. We explain such a difference by a combination of several factors – the differences in ester bond density, glass transition temperature, and crystallinity (surface crystallinity). The ratio of reactive ester groups to alkyl groups is the highest for PLLA and the lowest for PCL. Additionally, in the case of PCL, glass transition temperature (−60 °C) is far below the temperature of aminolysis, which provides high chain mobility and could result in free amine recombination reducing effective concentration of amine groups. Considering crystallinity, it is well known that the availability of ester groups for aminolysis is lower for the crystal phase with a dense package of molecules, hindering the diffusion of diamines.^57^ In our previous work, we showed differences in bulk crystallinity of these polyester fibers being the highest for PCL and the lowest for PLLA, together with a hypothesis of an additional factor related to the radial distribution of fiber structure with the more crystalline surface, which can hinder additionally aminolysis reaction for PCL.^17^ Contrary to some other works, a decrease of the amount of attached NH_2_ groups after a certain time threshold was not observed in our study.^43,45^

XPS results clearly indicate that aminolysis covers the entire volume of the fiber and not only the fiber's surface. Such conclusion can be drawn considering that the atomic concentration of the N–C/H bond is stable up to the depth of the 370 nm for the PLCL_10%_15 min. For the PLCL_Control, the measured carbon and oxygen content is close to the stoichiometric composition of the polymer, whose atomic concentration ratio C/O should be equal to 2.0. Relative intensities of the C–O and C=O bonds are also consistent with the stoichiometric composition of the analyzed compound. Differences observed for sample after aminolysis can be a result of the change of group orientation as aminolysis lead to chain scission.

Fragmentation of fibers that we observed for extreme aminolysis reaction conditions was explained by Polini A. *et al.* and Kim T. G. *et al.* as a result of chains scission, additional crystallization of shortened chains, and then the formation of a specific arrangement of alternate amorphous and crystalline domains.^58,59^ According to the authors, transverse fiber fragmentation can be caused by the easy breaking down of amorphous spaces present between crystalline regions under stress conditions.^58^ In our case, fragmentation could be favoured by sample handling. In the literature, there are many studies in which this fragmentation is desirable as aminolysis or other techniques are used for producing fibrous “particles”, especially from the PLLA nonwovens, that can be exploited as hydrogel fillers.^60–63^

In the literature, aminolysis is as an example of a method that improves the wettability (WCA) of material due to the hydrophilic character of amine groups.^46^ However, studies on aminolysis show that sometimes there is a lack of wettability improvement or changes are not significant.^17,49,64^ It was proposed by Monnier A. *et al.* that such a lack of improved wettability could be associated with the domination of the hydrophobic alkyl chain of diamine over the hydrophilic amine group.^64^ In our study, we have not observed a clear trend in the change of the WCA values. It should also be taken into consideration that WCA results are affected by roughness.^65^

The results of GPC analysis indicate clearly that the whole fiber, not only its surface, underwent an aminolysis reaction, which corresponds to the XPS analysis data. In chromatograms, we observed only one peak with its gradual shifting to the lowest molecular weight for all aminolyzed samples. In the case of reaction limited to the surface, a new peak for lower molecular weights would be expected to appear in the chromatogram in addition to the peak related to the fibers' unchanged interior. This is consistent with some fundamental studies showing that aminolysis, contrary to hydrolysis, is not a surface-limited reaction.^66,67^ There are also other papers, in which authors presented a significant decrease in molecular weight after aminolysis.^68,69^ However, as it was shown in our study, aminolysis of both, PCL and PLCL, allows obtaining relatively high amine concentration at the surface, maintaining a still reasonable level of molecular weight.

As it was proposed by Flory, the reduction of number average molecular weight, 
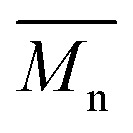
, leads to a decrease of fracture strength, *σ*, according to the equation:*σ* = *σ*^∞^ − *B*/*M*_n_,where *σ*^∞^ is the fracture strength at infinite molecular weight, and *B* is a constant dependent on chains' entanglements.^70^ In our study, in the case of PLCL sample with a significant reduction of 
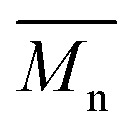
, a noticeable drop of stress at break was observed. This is not the case of PCL and PLLA, for which there is practically no change or even increase of stress at break. Additional factor for stress at break reduction in the case of PLCL is breaking and cracking of fibers due to aggressive aminolysis conditions. Another factor affecting mechanical properties is the additional crystallization caused by the presence of shorter, more mobile chains. This effect is most important for PLLA with large degradation leading to essential crystallization from practically amorphous state to crystallinity 50% in the case of PLLA_6%_10 min. In general, an increase of crystallinity is expected to be responsible for increase of stress at break, leading to disturb the correlation of stress at break with 
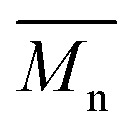
, as observed by us. Evident reduction of elongation at break with reduction of 
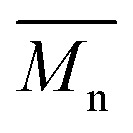
, can be explained by the loss of elastic properties with reduction of molecular entanglement density at lower molecular weight.

In the case of all aminolyzed samples, even with a lower level of attached amine groups, change of L929 shape to more spread was noticed. The same effect was observed for MG63 cells cultured on PCL and PLCL samples. Most probably, it is related to the positive charge of introduced amine groups as it was reported previously.^4,71^ Especially in the case of PLCL_10%_10 min, a lot of well-spread L929 cells with the formed filopodia were observed, which could be additionally associated with the fact that this sample was completely hydrophilic. Despite the improvement of cell morphology, results of metabolic activity tests did not show a clear correlation with the presence of amine groups for all polymers. Indeed, in the case of PCL, gradual improvement was observed, and taking into account that all PCL samples were hydrophobic, it is most probably associated with the effect of the positively-charged surface. However, general observations lead to the conclusion that introducing amine groups on the surface of the fibers leads in all cases to the proper cells morphology but is not sufficient for improvement of their metabolic activity. In other words, there is no clear correlation between improvement in cells morphology and their metabolic activity. There is little literature supporting these observations. For instance, Shin J. W. *et al.* show that 4-fold changes in scaffold stiffness, provoked by changes in the molecular structure, increase cellular spreading without significant impact on cell number and cellular viability.^72^ Despite many scientific inquiries, there is still no clear relation between cell viability, metabolic activity, and shape in the literature.^73^ We suppose that the observed SEM and FM morphology of cells is mostly affected by the quality of cellular adhesion to the fibers while the metabolic activity reflects further, post-adhesion stages of cells behaviour associated with their growth, migration and proliferation.

## 5. Conclusions

In this study, the aminolysis of three types of aliphatic polyester fibers in a wide range of conditions was investigated. It was shown that achieving the same level of amine concentration requires a much higher concentration of diamine and time of reaction in the case of PCL electrospun fibers compared to PLCL and PLLA. This phenomenon can be explained by the difference in ester bonds density, crystallinity (including surface crystallinity), and glass transition temperature. Despite the hydrophilic nature of amine groups, most of the samples remained hydrophobic. X-ray photoelectron spectroscopy results clearly demonstrate that an aminolysis reaction is not limited to the surface of the material. This observation is supported by GPC results showing bulk molecular weight decrease, which indicates that the reaction is not limited to the fiber surface, but it proceeds in the whole cross-section of the fiber.

Molecular degradation is much stronger for PLLA than for PLCL and PCL, leading to the favorable situation that for PCL and PLCL an effective concentration of amine groups is achieved at relatively weak degradation. Another effect of aminolysis is an increase of crystallinity as a result of shortening the chains and their additional arrangement. A decrease of molecular weight, as well as an increase of crystallinity, led to the change of mechanical properties. For the strongest treated samples, cracking and fragmentation of fibers were observed, affecting additional mechanical properties. However, our study shows that it is possible to reach a compromise between the concentration of introduced amine groups and change of fibers properties. Amine groups introduced in two different concentrations caused improvement of L929 and MG63 cells morphology to more spread. Taking into account that the low concentration of NH_2_ groups on the fiber surface was sufficient to improve the morphology of cells allowing at the same time to maintain relatively high molecular weight and hence mechanical strength, we recommend using aminolysis conditions that are relatively mild for a particular type of fibrous polymer scaffold.

## Conflicts of interest

There are no conflicts to declare.

## Supplementary Material

RA-012-D2RA00542E-s001
