# Supplementary material for: Aminolysis as a surface functionalization method of aliphatic polyester nonwovens: impact on material properties and biological response
Source: RSC Adv. 2022 Apr 11;12(18):11303–17. doi: 10.1039/d2ra00542e (PMC8997583; doi:10.1039/d2ra00542e)
Supplement: RA-012-D2RA00542E-s001 [file RA-012-D2RA00542E-s001.pdf]

Electronic Supplementary Material (ESI) for RSC Advances.
This journal is © The Royal Society of Chemistry 2022

Supplementary data:

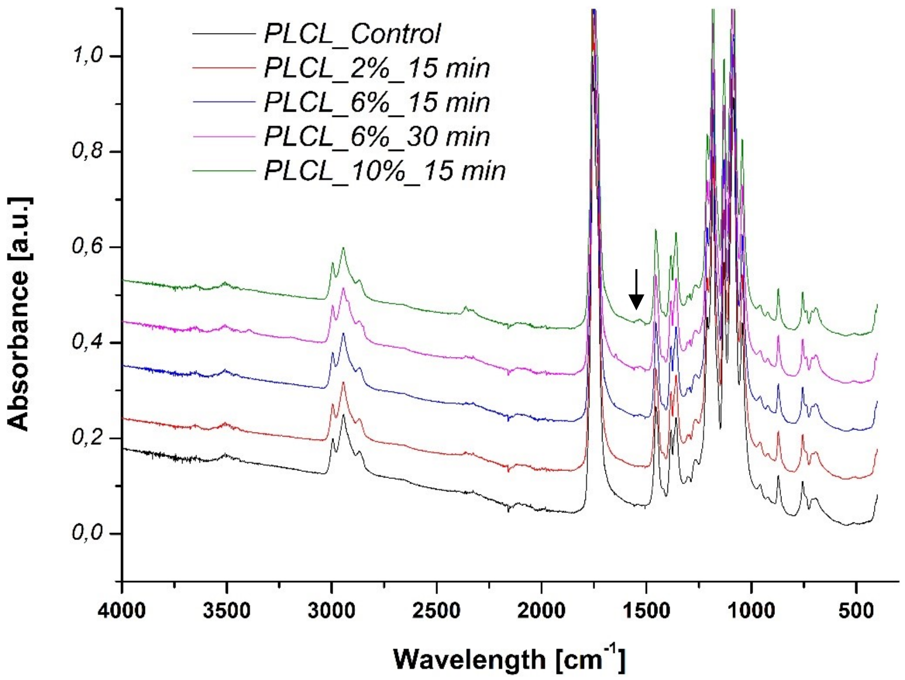

Figure S1. ATR-FTIR spectra of PLCL_Control and chosen PLCL samples after aminolysis with marked amide II peak.

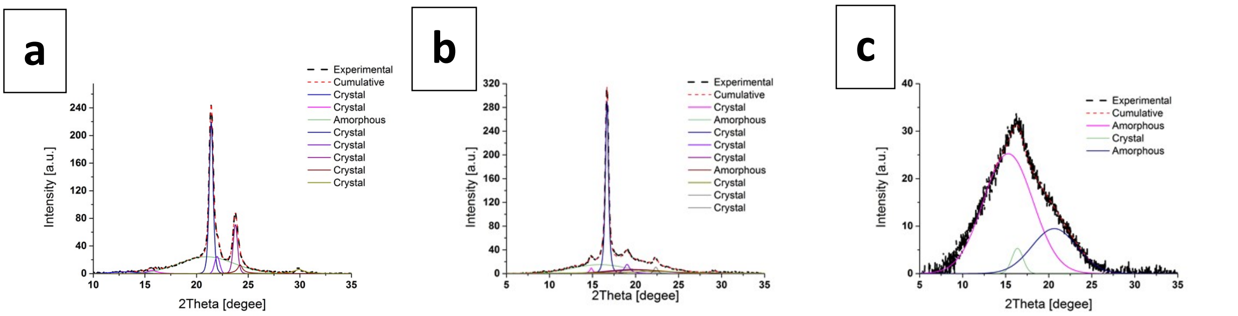

Figure S2. WAXS scattering profiles of: a) PCL_Control, b)PLCL_Control and c) PLLA_Control with fitting.

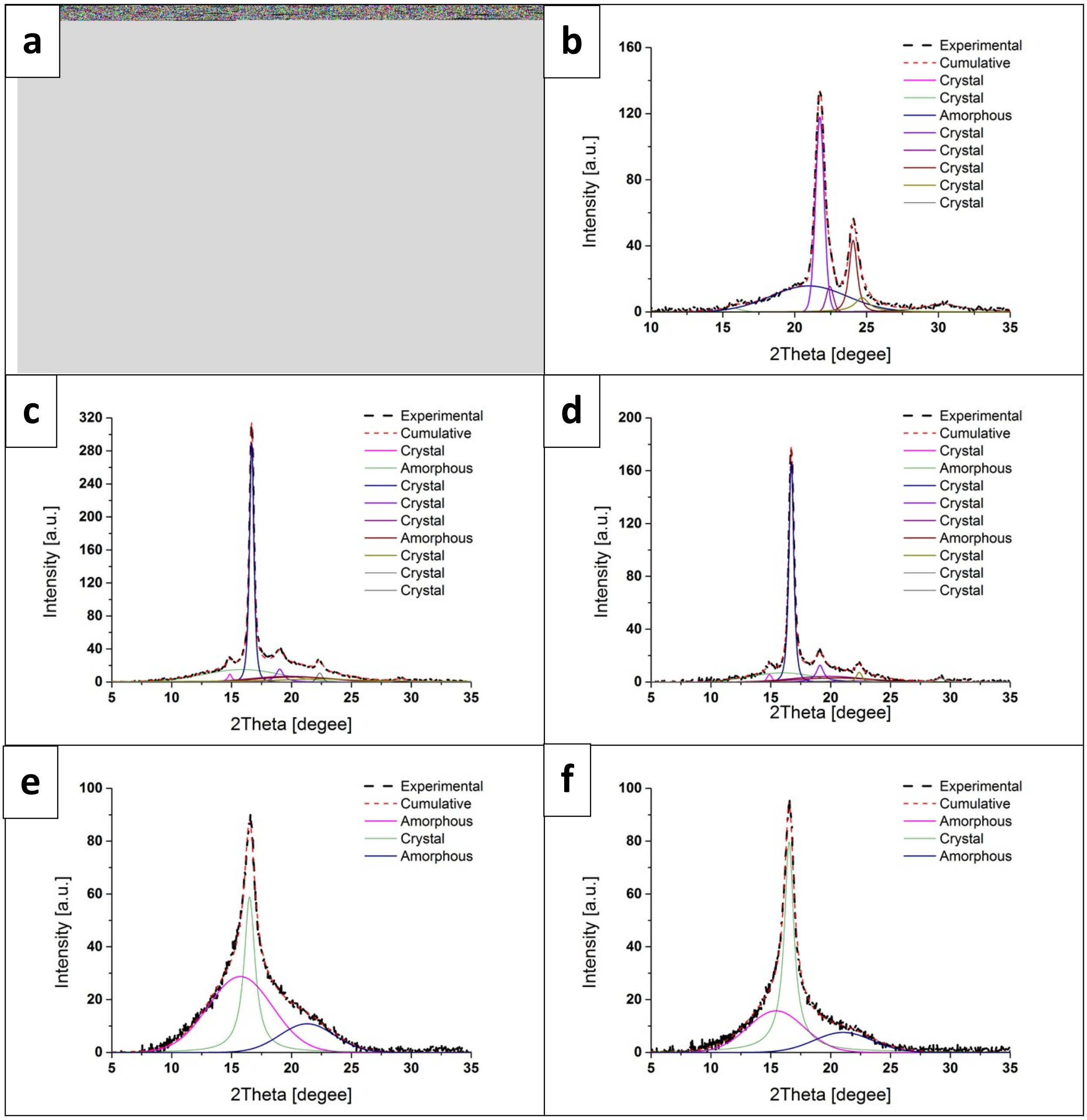

Figure S3. WAXS scattering profiles of: a) PCL_10%_24h, b) PCL_30%_24h, c) PLCL_2%_5 min, d) PLCL_10%_10 min, e) PLLA_2%_5 min, f) PLLA_6%_10 min with fitting.
